# No Interactions of Stacked Bt Maize with the Non-target Aphid *Rhopalosiphum padi* and the Spider Mite *Tetranychus urticae*

**DOI:** 10.3389/fpls.2018.00039

**Published:** 2018-02-02

**Authors:** Yinghua Shu, Jörg Romeis, Michael Meissle

**Affiliations:** ^1^Research Division Agroecology and Environment, Agroscope, Zurich, Switzerland; ^2^Department of Ecology, College of Natural Resources and Environment, South China Agricultural University, Guangzhou, China; ^3^Key Laboratory of Agro-Environment in the Tropics, Ministry of Agriculture, South China Agricultural University, Guangzhou, China

**Keywords:** Bt corn, Cry protein, SmartStax^®^, plant-insect-interactions, food web, arthropods, environmental risk assessment, non-target organism (NTO)

## Abstract

In the agroecosystem, genetically engineered plants producing insecticidal Cry proteins from *Bacillus thuringiensis* (Bt) interact with non-target herbivores and other elements of the food web. Stacked Bt crops expose herbivores to multiple Cry proteins simultaneously. In this study, the direct interactions between SmartStax^®^ Bt maize producing six different Cry proteins and two herbivores with different feeding modes were investigated. Feeding on leaves of Bt maize had no effects on development time, fecundity, or longevity of the aphid *Rhopalosiphum padi* (Hemiptera: Aphididae), and no effects on the egg hatching time, development time, sex ratio, fecundity, and survival of the spider mite *Tetranychus urticae* (Acari: Tetranychidae). The results thus confirm the lack of effects on those species reported previously for some of the individual Cry proteins. In the Bt maize leaves, herbivore infestation did not result in a consistent change of Cry protein concentrations. However, occasional statistical differences between infested and non-infested leaves were observed for some Cry proteins and experimental repetitions. Overall, the study provides evidence that the Cry proteins in stacked Bt maize do not interact with two common non-target herbivores.

## Introduction

As an alternative to chemical insecticides, genetically engineered (GE) crops producing insecticidal Cry proteins from the bacterium *Bacillus thuringiensis* (Bt) have been developed. Cry proteins are known to have a relatively specific range of biological activity. Cry1 and Cry2 proteins exhibit activity for the larvae of some species of butterflies and moths (Lepidoptera) while Cry3 and Cry34Ab1/Cry35Ab1 proteins are active against larvae of some leaf beetles (Coleoptera: Chrysomelidae). To broaden the target spectrum, to delay the evolution of resistance in the target pest(s), and to simplify crop management, multiple Cry proteins have been combined into modern GE plants. SmartStax^®^ maize produces the most combined GE traits of any currently commercially cultivated maize product, six different *cry* genes and two genes for herbicide tolerance (Head et al., 2017).

When insecticidal GE plants are grown in the field, the expressed Cry proteins may also interact with non-target herbivores and other components of the food web. Even though the Cry proteins engineered into Bt plants are known to be highly specific to Lepidoptera and Coleoptera, one concern with stacked Bt plants is that the different Cry proteins may interact synergistically and lead to unexpected non-target effects that are not observed when the individual Cry proteins are tested (Hilbeck and Otto, 2015). Potential interactions of different insecticidal proteins have been addressed in risk assessment studies using purified proteins and pest species that are sensitive to at least some of the used proteins. Graser et al. (2017) for example demonstrated that three different lepidopteran-active Cry proteins (Cry1Ab, Cry1F, Vip3A) acted in an additive way in tests with lepidopteran larvae. However, two coleopteran-active proteins (eCry3.1Ab, mCry3A) showed possible slight antagonism in tests with a coleopteran species. No effects of the coleopteran-active proteins were observed on the potency of the lepidopteran-active proteins against lepidopteran larvae and *vice versa*. This confirms earlier studies demonstrating no synergistic effects of the lepidopteran-active Cry1Ab and the coleopteran-active mCry3A (Raybould et al., 2012), of the lepidopteran-active Cry1Ac and Cry2Ab2 with another lepidopteran-active Bt protein, Vip3Aa19 (Levine et al., 2016), and of the coleopteran-active Cry3Bb1 with dsRNA used for an RNA-interference mode of action (Levine et al., 2015). While those studies used species that were sensitive to one of the insecticidal proteins and purified compounds, our study aimed at exploring potential effects of Bt maize producing several Cry proteins on non-target herbivores. We selected two non-target herbivores with different feeding modes: the aphid *Rhopalosiphum padi* (Hemiptera: Aphididae) and the spider mite *Tetranychus urticae* (Acari: Tetranychidae). Both species have a global distribution and are frequent pests of maize. While spider mites suck out mesophyll cells destructively, aphids feed on plant phloem. In consequence, spider mites ingest high amounts of Cry proteins, while aphids ingest only trace amounts, even when multiple Cry proteins are present (Svobodová et al., 2017). Previous laboratory studies with Bt maize producing single Cry1A proteins have shown no detrimental effects on the aphids *R. padi* (Dutton et al., 2002; Lumbierres et al., 2004), *Rhopalosiphum maidis* (Faria et al., 2008), and *Sitobion avenae* (Ramirez-Romero et al., 2008). Similarly, *T. urticae* was not affected when fed with maize producing Cry1Ab (Lozzia et al., 2000; Dutton et al., 2002), Cry1F (Guo et al., 2016), and Cry3Bb1 (Li and Romeis, 2010).

Based on the previously described results, the first hypothesis of the current study was that stacked Bt maize does not affect non-target herbivore performance compared to a genetically near non-Bt maize line. No data on potential effects of stacked Bt maize on spider mites and aphids are available and interaction studies of Cry proteins were done with sensitive target species in artificial diet systems using purified Cry proteins. Using SmartStax^®^ maize, our study thus evaluated experimentally, if six different Cry proteins, some of which have not been tested on aphids and spider mites previously, may lead to unexpected interactions and effects on non-target species when produced in the plant context. Furthermore, *in planta* studies might also reveal potential indirect (plant-mediated) effects due to the transformation process and the plant’s physiological responses (Ladics et al., 2015).

In addition to potential effects that Bt plants might have on non-target herbivores, the feeding of herbivores might also affect the plant. From the perspective of plant defense, herbivores could potentially influence the production of Cry proteins in the plant. Prager et al. (2014) claimed that the Cry1Ab and Cry3Bb1 concentrations in stacked Bt maize was reduced when plants were infected with spider mites. Such an effect could have important implications as it could lower the efficacy of the Bt plant against target herbivores. However, no such effects on Cry protein concentrations were observed with Bt cotton producing Cry1Ac and Cry2Ab and Bt maize producing Cry1F (Guo et al., 2016).

The second hypothesis of the current study thus was that herbivore infestation does not affect Cry protein concentrations in stacked Bt maize compared to non-infested Bt maize. To test this hypothesis, we worked with SmartStax^®^ maize and two herbivores, *R. padi* and *T. urticae*. Using commercial ELISA kits, we measured five of the six plant-produced Cry proteins. This combination of Cry proteins and herbivores might reveal general patterns of herbivore effects on Cry protein expression if present.

## Materials and Methods

### Maize Plants

Stacked Bt maize (Genuity^®^ SmartStax^®^, event MON89034 × TC1507 × MON88017 × DAS-59122-7, Monsanto Company, St. Louis, MO, United States) and the nearest conventional non-Bt hybrid (EXP 258, Monsanto) were used for all experiments. SmartStax^®^ expresses genes for the Lepidoptera-active Bt proteins Cry1A.105, Cry1F, and Cry2Ab, the Coleoptera-active proteins Cry3Bb1, Cry34Ab1, and Cry35Ab1, and two herbicide tolerance genes. Plants were grown individually in 12 L plastic pots and were fertilized with 40 g of slow release fertilizer (Manna, Wilhelm Haug GmbH, Ammerbuch, Germany) before sowing and weekly with 0.2–0.8 L of 0.2% liquid NPK fertilizer (Manna, Wilhelm Haug GmbH).

For the herbivore performance assays, Bt and non-Bt maize was grown in a climatic chamber (25 ± 1°C, 16:8 h L:D regime, 75 ± 5% humidity) and used when 9–10 leaves were expanded (approximately 4–5 weeks old plants). For the Cry-protein experiments, Bt maize was grown in a glasshouse (approximately 25°C) and used in the 10 leaves stage (5 weeks old).

### Arthropods

*Rhopalosiphum padi* aphids and *T. urticae* spider mites were used from our own cultures on maize plants, which were started with individuals supplied by Syngenta Crop Protection Münchwilen AG (Stein, Switzerland). Both species were reared on Bt and non-Bt maize in the same climate chamber, but spatially separated to limit exchange between the two treatments.

### Aphid Performance on Bt Maize

The experiment was conducted twice with 13 and 16 plants per maize treatment in the first and second run, respectively, resulting in a total of 29 plants per treatment. Bt and non-Bt plants were distributed alternately in blocks of 3–4 plants in the climate chamber. Two reproductive aphids from the culture were settled on the 5th, 6th, and 7th leaf and covered with one transparent plastic clip cage (3.5 cm diameter; 1 cm height) per leaf, resulting in a total of 39 and 48 clip cages per treatment in the first and second experimental run, respectively. Aphids for Bt maize or non-Bt maize were collected from the respective maize plants in the culture. Clip cages had a hole sealed with fine-mesh netting to provide air-circulation and foam rubber rings to gently seal against the leaf. In the first run of the experiment, all aphids except one neonate nymph were removed after 24 h (defined as day 0). Nymphs that were not sitting on the leaf were replaced by neonate nymphs within the following 2 days (unsuccessful settlement). The number of nymphs that needed to be replaced in this way was relatively high, but treatment-independent (20 in the Bt and 20 in the non-Bt treatment). In the second run of the experiment, the protocol was changed and 2–3 neonate nymphs were left on the leaves on day 0. After 24 h, the surplus nymphs were removed so that only one nymph remained in each clip cage. In this way, no nymphs needed to be replaced. Every day, aphid survival and the number of offspring produced by each aphid after reaching adulthood were recorded and neonate nymphs were removed. Aphids were monitored until death.

The nymphal development time was defined as the number of days from day 0 to the day when first offspring was observed. Adult longevity was calculated from the day when first nymphs were observed to the day the adult was found dead. Total fecundity was the sum of all offspring produced by an aphid during the experiment.

### Spider Mite Performance on Bt Maize

The experiment was conducted twice with 15 plants per maize treatment in each run, resulting in a total of 30 plants per treatment. Bt and non-Bt plants were distributed alternately in blocks of 3–4 plants in the climate chamber. Spider mites were kept on leaf disks according to Li and Romeis (2010). Round leaf disks (ca. 2.5 cm diameter) were cut from the 5th, 6th, and 7th leaf of each plant and kept in a sandwich of two cotton pads. A hole (ca. 1 cm diameter) was cut in the middle of the upper pad. The cotton pads were wetted with tap water and kept in transparent plastic dishes (5 cm diameter, 1 cm height) covered with a ventilated lid. Using a binocular microscope, one female spider mite was transferred from the culture to each leaf disk using a fine paint brush with only 1 hair. Spider mites for Bt or non-Bt treatments were collected from the respective maize plants in the culture. The leaf disks were stored in a climate cabinet (MLR-352H-PE, Panasonic Biomedical, Etten-Leur, The Netherlands) at 25 ± 1°C, 16:8 h L:D regime, and 75 ± 5% relative humidity. Next day, the females were removed and all eggs except one were destroyed with a needle (defined as day 0). In the following, the cotton pads were rewetted, the hatching, survival, development, and reproduction of the spider mites were recorded, and the eggs were destroyed daily. Spider mites were transferred to new leaf disks every 3–4 days to ensure constant quality of food and exposure to the Cry proteins (Zurbrügg and Nentwig, 2009). New disks were cut next to the holes from the previous disks from the same leaves. The transfer of immobile larvae and nymphs (in the process of molting), was postponed to the next day. Once a male hatched from the deuteronymph, the experiment ended for this individual. In each cage with a newly hatched female, two (run 1) or one (run 2) male from the experiment or the culture was added to ensure mating. Only one male was used in the second run of the experiment, because mortality of males was very high in the first run, most likely because of competition among the two males. Males were removed after 3 days. Within those days, however, dead males were replaced. Egg fertility was examined in the second run of the experiment. When leaf disks were changed, the old leaf disks with eggs were incubated for 5–6 days and the number of unhatched eggs was counted and compared with the total number of eggs on the disk.

Egg hatching time was defined as the number of days from day 0 to the day when the larva hatched from the egg. Nymphal development time was the number of days from the day when the larva hatched to the day when the adult emerged. Female longevity was calculated from the day when the female emerged to the day it was found dead. Total fecundity included all eggs laid by a female during the experiment. Sex ratio was defined as the percentage of females.

### Cry Protein Content in Maize after Aphid Infestation

Bt maize plants were grouped in six blocks of 3–4 plants in a large glasshouse cabin. The experiment was conducted twice with 20 and 22 plants in the first and second run, respectively. Before the plants were infested with aphids, four leaf disks (0.5 cm diameter) were punched from the middle part of the 9th leaf (counted from the base) using a common office hole-punch, placed in a 2 ml microtube and frozen at -80°C. The plants in half of the blocks (10 and 12 plants in the first and second run, respectively) were infested with aphids (approximately 100 aphids per plant), while the plants in the other half of the blocks remained non-infested. During the experiment, the non-infested plants were controlled daily and incidental aphids were removed. The experiment ended after 20 days, when infested plants were heavily populated with aphids. At the end of the experiment, four leaf disks (0.5 cm diameter) were sampled as described previously from the same leaf that had been sampled at the start of the experiment (9th leaf) and additionally from the first fully expanded leaf at the top (14th or 15th leaf).

### Cry Protein Content in Maize after Spider Mite Infestation

Spider mites are much smaller than aphids, move through the air by ballooning, and it is practically impossible to control plants for unintended spider mite infestation. Therefore, infested and non-infested plants had to be kept in separate glasshouse cabins. The experiment was conducted twice with 24 and 26 plants in the first and second run, respectively. The cabins were switched for the non-infested and infested treatments for the two runs. As described previously, four leaf disks were sampled from the 9th leaf before spider mite infestation. The plants in one cabin were infested with several 100 spider mites per plant, while the plants in the other cabin remained non-infested. The experiment ended after 20–22 days. Leaf disks were sampled once more from the 9th leaf and also from the first unfolded leaf at the top (14th or 15th leaf). At that time, leaves in the lower part of the plant were heavily damaged by spider mites, while the youngest leaves showed no (run 1) or little (run 2) damage. To document leaf damage, photos were taken with a Leica microscope/camera system (see Supplementary Figure S1). The climatic similarity of the two glasshouse cabins was confirmed with data loggers (Elpro Ecolog, Elpro-Buchs AG, Buchs, Switzerland), which revealed that the difference in mean temperature over the experimental period was smaller than 0.5°C and 15% RH.

### Quantification of Cry Proteins

All collected leaf samples were lyophilized and weighed. Cry proteins were quantified individually with commercial double antibody sandwich (DAS) ELISA kits for Cry1Ab-1Ac (used to detect Cry1A.105), Cry1F, Cry2A, Cry3Bb1, and Cry34Ab1 (Agdia, Inc., Elkhart, IN, United States). The concentrations of the different Cry proteins were estimated as described by Svobodová et al. (2017) with the exception that standard curves were based on a single rectangular hyperbola model (SigmaPlot 13.0, Systat Software, Inc.). Leaf samples had to be diluted to achieve concentrations within the measurement range of the individual kits. If a large number of samples in one treatment was below the limit of detection or in the plateau area of the standard curve, all samples of the respective experiment were analyzed again for the respective Cry protein using a more appropriate dilution.

### Data Analysis

All data used for statistical analysis, tables and figures for this publication can be found in the Supplementary Material online (**Supplementary Data Sheet 1**). Data from the herbivore performance assays were analyzed with linear or generalized linear models (GLMs) using R statistical software (R version 3.1.0, The R Foundation for Statistical Computing, Vienna, Austria). For all analyzed factors, contrasts were set to orthogonal. Applied models were full factorial for fixed factors.

Nymphal development time and adult longevity of both herbivore species were analyzed by generalized linear mixed-effects models (GLMMs) with Poisson distribution from the lme4 package. Fixed effects were treatment (Bt or non-Bt), leaf (5th, 6th, or 7th), and run (1, 2) and random effect was plant. Effects of factor and interactions were determined from an ANOVA table with Type III sum of squares (car package). Total fecundity of both species was analyzed similarly with linear mixed-effects models (LMM) from the lme4 package.

While the influence of the factors plant and leaf on spider mite egg fitness (which might affect hatching time) and sex were most likely minimal (predetermined by the female from the culture), run and treatment might have had an effect because spider mites were reared for several generations on Bt or non-Bt plants and the experiment was conducted at different points in time. Therefore, egg hatching time and sex ratio were analyzed by GLMs with the fixed factors treatment and run, a Poisson distribution for egg hatching time, and a binomial distribution (Logit link function) for sex ratio.

Total longevity of both species was analyzed among the Bt and non-Bt treatment with pooled data (no separation of other variables) (package survival). Censored data included all males of spider mites after emergence, and damaged and lost individuals of both species. The treatments were compared with a log-rank test in the survdiff function.

Power analyses were performed to determine the detectable differences (percentage difference of detectable treatment means relative to control means) based on the means and SEs of the non-Bt treatment (package pwr). For time and fecundity data, effect size d was calculated based on two-sided t-tests, the true sample size (N) of the non-Bt treatment, a power of 80%, alpha-level of 5%, and assuming equal sample sizes. Using d, a hypothetical second mean (mean_hypo_) was calculated from the mean and the SD of the non-Bt group based on the equation (Cohen, 1988):

$$\text{d}=({\text{mean}}_{\text{non}-\text{Bt}}-{\text{mean}}_{\text{hypo}})/{\text{SD}}_{\text{non}-\text{Bt}}$$

The detectable difference was then calculated from the proportions of both means:

$$\text{det}.\text{diff}=1-({\text{mean}}_{\text{hypo}}/{\text{mean}}_{\text{non}-\text{Bt}})$$

For the sex ratio, effect size h was similarly calculated based on a test of two proportions. A hypothetical second proportion was calculated with h and the proportion of females in the non-Bt treatment (p_non-Bt_) based on the equation (Cohen, 1988):

h=2arcsin(√p1)−2arcsin(√p2)

The equation was once solved for p2 with p1 = p_non-Bt_ and once for p1 with p2 = p_non-Bt_. The detectable difference was then the difference between p1 and p2 and an average of both calculations (equation solved for p2 or p1) was reported.

Because ELISA estimates of Cry protein concentrations were highly variable and variances were inhomogeneous, they were analyzed visually based on 95% confidence intervals around the means. Significant differences between herbivore-infested and non-infested plants were concluded from non-overlapping confidence intervals.

## Results

### Herbivore Performance Tests

Individual aphids developing in clip cages on leaves of Bt and non-Bt maize in the climate chamber had a similar nymphal development time, adult longevity, total fecundity, and total longevity (**Table 1**). Individual spider mites developing on leaf disks from Bt and non-Bt maize had a similar egg hatching time, nymphal development time, female longevity, sex ratio, total fecundity, and total longevity (**Table 1**). The experimental repetition (run) had a significant effect on all measured parameters in the aphid experiment and on total fecundity and female longevity in the spider mite experiment (Supplementary Table S1). The leaf where clip cages were positioned or from which leaf disks were cut (5th, 6th, or 7th) had no effect on any of the measured parameters (Supplementary Table S1).

**Table 1:** Life table parameters of herbivores fed with SmartStax^®^ maize (Bt) or the nearest conventional line (non-Bt).

	Mean ± SE (N)			
Parameter	Bt	Non-Bt	*p*-value	Detectable difference (%)
*Rhopalosiphum padi*				
Nymphal development time (d)	6.1 ± 0.14 (83)	5.9 ± 0.13 (86)	0.40	9
Adult longevity (d)	17.9 ± 0.80 (74)	17.9 ± 0.77 (75)	0.97	17
Total fecundity (no. of nymphs)	52.3 ± 2.65 (78)	54.0 ± 2.65 (84)	0.48	20
Total longevity (d)	24.1 ± 0.80 (89)	23.9 ± 0.85 (92)	0.85	–
*Tetranychus urticae*				
Egg hatching time (d)	4.0 ± 0.03 (89)	3.9 ± 0.04 (87)	0.82	4
Nymphal development time (d)	5.7 ± 0.09 (84)	5.8 ± 0.08 (80)	0.74	6
Sex ratio (% females)	60.7 ± 5.36 (84)	66.7 ± 5.27 (81)	0.35	20
Female longevity (d)	10.4 ± 0.81 (48)	10.1 ± 0.66 (51)	0.90	26
Total fecundity (no. of eggs)	81.3 ± 7.53 (48)	86.2 ± 7.30 (51)	0.78	34
Total longevity (d)	19.8 ± 0.79 (89)	19.4 ± 0.70 (87)	0.51	–

*Results of statistical tests are provided (p-values). More details on statistics can be found in Supplementary Table S1. Power analyses were performed to determine the detectable differences based on proportions for sex ratio and t-tests for all other parameters, a power of 80%, an alpha-level of 5%, and the means and SDs of the non-Bt treatment assuming equal sample sizes and two-sided tests. N = number of replicates.*

The observed non-Bt means and standard deviations and the true sample sizes were used to calculate the detectable differences, i.e., the percentage difference of detectable treatment means relative to control means (α = 0.05, power = 0.8). The lowest detectable differences (<10%) were observed for egg hatching time of spider mites and nymphal development time of aphids and spider mites (**Table 1**). Highest detectable differences of 20 and 34% were revealed for total fecundity of aphids and spider mites, respectively.

Egg fertility in the spider mite assay was high. Only 2.3 and 3.6% of the eggs in the non-Bt and Bt treatment, respectively, did not hatch.

### Cry Protein Content after Herbivore Infestation

**FIGURE 1:**
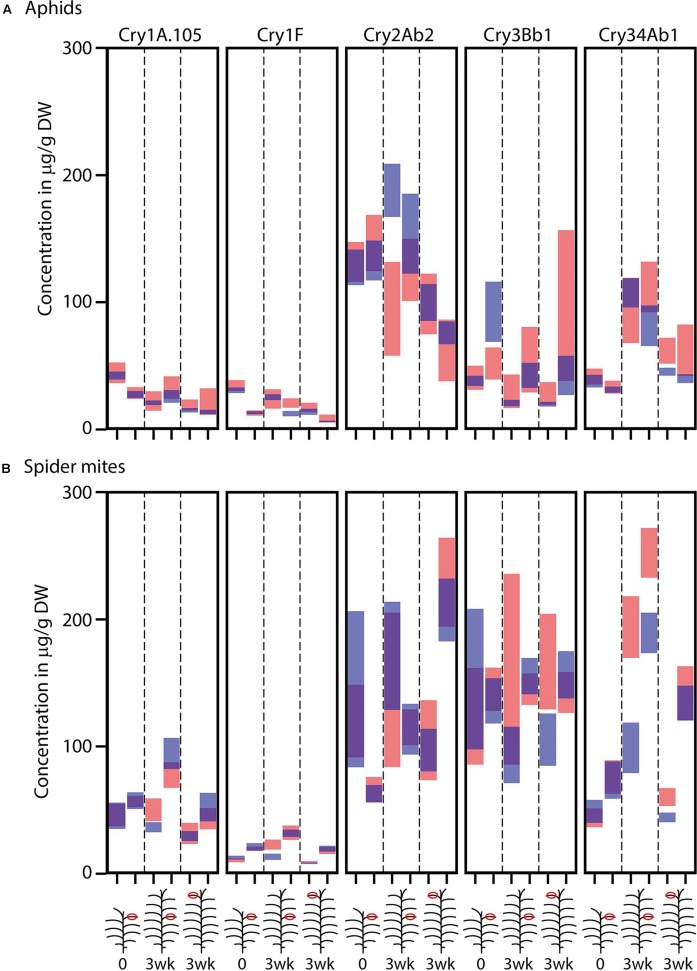
Estimated Cry protein concentrations (μg/g dry weight) in leaves of SmartStax^®^ maize before and after infestation with **(A)** aphids (*Rhopalosiphum padi*) or **(B)** spider mites (*Tetranychus urticae*). Samples taken from the 9th leaf counted from the bottom before infestation are labeled with 0, samples taken 3 weeks later are labeled with 3 wk. The maize illustrations indicate if samples were taken from the 9th leaf or from the 1st fully expanded leave at the top. Within each leaf/time category, the left and right bars represent the first and second run of the experiment, respectively. Each panel represents one Cry protein. Red bars indicate the 95% confidence intervals for the samples from the plants infested or foreseen to be infested with herbivores and blue bars indicate plants without herbivores. Overlapping confidence intervals have purple color. Detailed data can be found in Supplementary Table S2.

As anticipated, samples from the 9th leaf of Bt maize plants at time point 0 designated for herbivore infestation showed similar Cry protein concentrations to those designated to remain non-infested, based on overlapping 95% confidence intervals (**Figure 1** and Supplementary Table S2). One exception was Cry3Bb1 in the second run of the aphid experiment, where leaves of the non-infested treatment had significantly higher Cry3Bb1 concentrations than leaves from the designated infested plants.

After infestation with aphids, the 9th leaf showed a 40% higher concentration of Cry1F in the second run and a 50% lower concentration of Cry2Ab2 in the first run compared to non-infested leaves. The first fully expanded leaf at the top of aphid infested plants showed a 30% higher concentration of Cry34Ab1 in the first run compared to non-infested plants. After infestation with spider mites, the 9th leaf showed a higher concentration of Cry1A.105 and Cry1F in the first run and a higher concentration of Cry34Ab1 in both runs compared to non-infested leaves. The top leaf of spider mite infested plants showed a higher concentration of Cry3Bb1 and Cry34Ab1 in the first run compared to non-infested plants (**Figure 1** and Supplementary Table S2).

## Discussion

The performance assays with spider mites and aphids did not reveal any significant difference between the Bt and the non-Bt treatment for any of the measured life-table parameters. The power analysis (i.e., calculated detectable differences) provide information on the size of effects that could be detected with the given sample size and variation in the data, although those calculations, mainly based on simple *t*-tests, cannot be directly compared with the more complex statistical models used to determine differences between Bt and non-Bt treatments. The power analyses indicated that the smallest differences could be detected for nymphal development times (<10%), while parameters for adults (fecundity, longevity) were less sensitive (20–34%). This is not surprising because nymphal development was relatively uniform while adult survival and reproduction showed high variation. Similar detectable differences were reported for *T. urticae* feeding on maize by Li and Romeis (2010).

Aphids have been shown to ingest at most traces of Cry protein when feeding on Bt maize, as they feed on the phloem which does not contain high quantities of Cry proteins (Raps et al., 2001; Romeis and Meissle, 2011; Svobodová et al., 2017). In contrast, spider mites on SmartStax^®^ were found to contain Cry proteins in concentrations of the same order of magnitude as leaves (Svobodová et al., 2017), as expected given mites consume leaf tissue which corresponds to plant level exposure.

Independent from the exposure to Cry proteins, unexpected differences due to the transformation process or differences among the Bt and non-Bt comparator not related to the produced Cry proteins could occur. Such effects, however, have not been observed in the current study. Our results confirm those of previous studies with Bt maize producing single Cry proteins. Li and Romeis (2010) reported no differences in life-table parameters for *T. urticae* feeding on Cry3Bb1-producing Bt maize compared to mites feeding on non-Bt maize. Similarly, *T. urticae* survival, development and reproduction were not affected when feeding on Cry1F-producing maize (Guo et al., 2016) or Cry1Ab-producing maize (Lozzia et al., 2000). Dutton et al. (2002) reported similar intrinsic rates of natural increase (r_m_) for *T. urticae* and *R. padi* feeding on Cry1Ab-producing maize compared to non-Bt maize. Lumbierres et al. (2004) reported positive effects of Cry1Ab-producing Bt maize compared to the near isoline on the first generation of alate *R. padi* aphids, but opposite effects on the offspring of apterous mothers. The authors conclude that the observed differences in aphid development may be linked to changes in host-plant quality. Faria et al. (2008) did not observe differences in growth rate of individual *Rhopalosiphum maidis* aphids among six different pairs of Cry1Ab-producing Bt maize and their near-isolines. However, they observed more aphids on Bt maize in five of the six Bt/non-Bt maize pairs when large populations were left to develop for 5 weeks. Ramirez-Romero et al. (2008) reported no differences in developmental parameters of another aphid species, *Sitobion avenae*, when developing on Cry1Ab-producing Bt maize and its near-isoline.

One concern with stacked Bt crops is that the different Cry proteins may interact synergistically and result in adverse effects on the food web in a way not observed with plants producing only one Cry protein (Hilbeck and Otto, 2015). The present study, however, provides evidence that the different Cry proteins do not interact in a way that the two non-target herbivores are affected. Similarly, a companion study with the same plant material did not report any effects when Bt maize pollen and *T. urticae* and *R. padi* reared on Bt maize were fed to the predators *Chrysoperla carnea* (Neuroptera: Chrysopidae), *Phylloneta impressa* (Aranea: Theridiidae), and *Harmonia axyridis* (Coleoptera: Coccinellidae) (Svobodová et al., 2017). This supports the studies with sensitive herbivores that revealed no synergistic effects of different combinations of purified Cry proteins (Raybould et al., 2012; Levine et al., 2016; Graser et al., 2017) or Cry proteins with dsRNA (Levine et al., 2015).

Herbivore infestation had no consistent effect on Cry protein production by the plants. While in eight out of 40 comparisons, confidence intervals of Cry protein concentrations between infested and uninfested Bt maize plants did not overlap, these differences were only significant in one of two runs. One exception is Cry34Ab1 in the spider mite study, which was higher in the 9th leaf of infested plants in both runs (and in the top leaf in run 1). Higher Cry34Ab1 levels were also measured in top leaves of aphid infested plants, significant in the first run and almost in the second. While our results are in line with those reported by Guo et al. (2016) for Cry1F producing Bt maize infested with *T. urticae*, they are in contrast to Prager et al. (2014) who reported decreased levels of Cry1Ab and Cry3Bb1 when stacked Bt maize was infested with *Tetranychus cinnabarinus* spider mites at a comparable growth stage. No significant changes or elevated concentrations were evident with Cry1A.105 (although this protein might not be directly comparable to Cry1Ab) and Cry3Bb1 after infestation with spider mites in the present study.

The highly variable and sometimes very large confidence intervals demonstrate that ELISA estimates have to be interpreted with caution and should be regarded as semi-quantitative only. The commercial ELISA kits that we used are designed for qualitative detection of Cry proteins in leaves and our protocol for quantitative detection has not been truly validated. To ensure highest comparability among the treatments of one experimental run, we measured all samples from one Cry protein and one experimental run on the same plate. When several values of one Cry protein were out of range, the whole measurement (rather than individual samples) was repeated with a more appropriate dilution. Therefore, we are confident that the statistical comparisons that we made are based on comparable Cry protein concentration estimates. Repeated measurements for Cry1A.105 and Cry2Ab2 were generally in the same range as the first measurement, indicating that the additional freezing/thawing cycle did not degrade the Cry proteins to a large extent. In contrast, however, Cry3Bb1 showed ca. 80% lower values for the repeated samples and Cry1F showed 50% lower values. This clearly shows that values of the different Cry proteins and between experimental runs cannot be compared directly. Even more variation is likely when different protocols or different ELISA kit lots are used, thus comparing ELISA estimates across studies is cautioned (Nguyen et al., 2008).

Overall, the present laboratory and glasshouse studies provide evidence that the six Cry proteins (and two herbicide tolerance proteins) produced in SmartStax^®^ maize did not interact with the two non-target herbivores studied, i.e., *R. padi* and *T. urticae*. Similar to Bt maize expressing single Cry proteins, stacked Bt maize is unlikely to affect populations of these herbivores via direct exposure. The hypotheses that stacked Bt maize does not affect non-target herbivore performance compared to a genetically near non-Bt maize line and that herbivore infestation does not affect Cry protein concentrations in stacked Bt maize compared to non-infested Bt maize are supported.

## Author Contributions

All authors contributed to the planning and designing of the experiments and to the drafting of the manuscript. YS and MM performed the bioassays and ELISA analyses. MM analyzed the data statistically.

## Conflict of Interest Statement

The authors declare that the research was conducted in the absence of any commercial or financial relationships that could be construed as a potential conflict of interest.

## Supplementary Material

DATA SHEET S1 Raw data used for statistical analyses, tables and figures of this publication.

## References

[B1] Cohen J. (1988). *Statistical Power Analysis for the Behavioural Sciences*, 2nd Edn. Hillsdale NJ: Lawrence Erlbaum Associates.

[B2] Dutton A. Klein H. Romeis J. Bigler F. (2002). Uptake of Bt-toxin by herbivores feeding on transgenic maize and consequences for the predator *Chrysoperla carnea*. *Ecol. Entomol.* 27 441–447. 10.1046/j.1365-2311.2002.00436.x

[B3] Faria C. A. Wäckers F. L. Pritcherd J. Barrett D. A. Turlings T. C. J. (2008). High susceptibility of Bt maize to aphids enhances the performance of parasitoids of lepidopteran pests. *PLOS ONE* 2:e600. 10.1371/journal.pone.0000600 17622345 PMC1899225

[B4] Graser G. Walters F. S. Burns A. Sauve A. Raybould A. (2017). A general approach to test for interaction among mixtures of insecticidal proteins which target different orders of insect pests. *J. Insect Sci.* 17 39. 10.1093/jisesa/iex003 28355479 PMC5416900

[B5] Guo Y. Y. Tian J. C. Shi W. P. Dong X. H. Romeis J. Naranjo S. E. (2016). The interaction of two-spotted spider mites, *Tetranychus urticae* Koch, with Cry protein production and predation by *Amblyseius andersoni* (Chant) in Cry1Ac/Cry2Ab cotton and Cry1F maize. *Transgenic Res.* 25 33–44. 10.1007/s11248-015-9917-1 26545599

[B6] Head G. P. Carroll M. W. Evans S. P. Rule D. W. Willse A. R. Clark T. L. (2017). Evaluation of SmartStax and SmartStax PRO maize against western corn rootworm and northern corn rootworm: efficacy and resistance management. *Pest Manag. Sci.* 73 1883–1899. 10.1002/ps.4554 28195683

[B7] Hilbeck A. Otto M. (2015). Specificity and combinatorial effects of *Bacillus thuringiensis* cry toxins in the context of GMO environmental risk assessment. *Front. Environ. Sci.* 3:71 10.3389/fenvs.2015.00071

[B8] Ladics G. S. Bartholomaeus A. Bregitzer P. Doerrer N. G. Gray A. Holzhauser T. (2015). Genetic basis and detection of unintended effects in genetically modified crop plants. *Transgenic Res.* 24 587–603. 10.1007/s11248-015-9867-7 25716164 PMC4504983

[B9] Levine S. L. Mueller G. M. Uffman J. P. (2016). Assessing the potential for interaction between the insecticidal activity of two genetically engineered cotton events combined by conventional breeding: An example with COT102 × MON 15985. *Regul. Toxicol. Pharmacol.* 79 35–41. 10.1016/j.yrtph.2016.05.003 27155596

[B10] Levine S. L. Tan J. Mueller G. M. Bachman P. M. Jensen P. D. Uffman J. P. (2015). Independent action between DvSnf7 RNA and Cry3Bb1 protein in southern corn rootworm, *Diabrotica undecimpunctata* howardi and Colorado potato beetle, *Leptinotarsa decemlineata*. *PLOS ONE* 10:e0118622. 10.1371/journal.pone.0118622 25734482 PMC4348175

[B11] Li Y. Romeis J. (2010). Bt maize expressing Cry3Bb1 does not harm the spider mite, *Tetranychus urticae*, or its ladybird beetle predator, *Stethorus punctillum*. *Biol. Control* 53 337–344. 10.1016/j.biocontrol.2009.12.003

[B12] Lozzia G. C. Rigamonti I. E. Manachini B. Rocchetti R. (2000). Laboratory studies on the effects of transgenic corn on the spider mite *Tetranychus urticae* Koch. *Boll. Zool. Agr. Bachic. Ser. II* 32 35–47. 10.1007/s11248-015-9917-1 26545599

[B13] Lumbierres B. Albajes R. Pons X. (2004). Transgenic Bt maize and *Rhopalosiphum padi* (Hom., Aphididae) performance. *Ecol. Entomol.* 29 309–317. 10.1111/j.0307-6946.2004.00597.x

[B14] Nguyen H. T. Hunfeld H. Meissle M. Miethling-Graf R. Pagel-Wieder S. Rauschen S. (2008). Round robin quantitation of Cry3Bb1 using the qualitative PathoScreen ELISA. *IOBC-WPRS Bull.* 33 59–66.

[B15] Prager S. M. Martini X. Guvvala H. Nansen C. Lundgren J. (2014). Spider mite infestations reduce *Bacillus thuringiensis* toxin concentration in corn leaves and predators avoid spider mites that have fed on *Bacillus thuringiensis* corn. *Ann. Appl. Biol.* 165 108–116. 10.1111/aab.12120

[B16] Ramirez-Romero R. Desneux N. Chaufaux J. Kaiser L. (2008). Bt-maize effects on biological parameters of the non-target aphid *Sitobion avenae* (Homoptera: Aphididae) and Cry1Ab toxin detection. *Pest. Biochem. Physiol.* 91 110–115. 10.1016/j.pestbp.2008.01.010

[B17] Raps A. Kehr J. Gugerli P. Moar W. J. Bigler F. Hilbeck A. (2001). Immunological analysis of phloem sap of *Bacillus thuringiensis* corn and of the nontarget herbivore *Rhopalosiphum padi* (Homoptera: Aphididae) for the presence of Cry1Ab. *Mol. Ecol.* 10 525–533. 10.1046/j.1365-294x.2001.01236.x 11298965

[B18] Raybould A. Graser G. Hill K. Ward K. (2012). Ecological risk assessment for transgenic crops with combined insect-resistance traits: the example of Bt11 × MIR604 maize. *J. Appl. Entomol.* 136 27–37. 10.1111/j.1439-0418.2010.01601.x

[B19] Romeis J. Meissle M. (2011). Non-target risk assessment of Bt crops – Cry protein uptake by aphids. *J. Appl. Entomol.* 135 1–6. 10.1111/j.1439-0418.2010.01546.x

[B20] Svobodová Z. Shu Y. Skoková Habuštová O. Romeis J. Meissle M. (2017). Stacked Bt maize and arthropod predators – Exposure to insecticidal Cry proteins and potential hazards. *Proc. Royal Soc. B* 284 20170440. 10.1098/rspb.2017.0440 28724730 PMC5543214

[B21] Zurbrügg C. Nentwig W. (2009). Ingestion and excretion of two transgenic Bt corn varieties by slugs. *Transgenic Res.* 18 215–225. 10.1007/s11248-008-9208-1 18763046

