# Supplementary material for: No Interactions of Stacked Bt Maize with the Non-target Aphid Rhopalosiphum padi and the Spider Mite Tetranychus urticae
Source: Front Plant Sci. 2018 Feb 2;9:39. doi: 10.3389/fpls.2018.00039 (PMC5801577; doi:10.3389/fpls.2018.00039)
Supplement: Supplementary file 2 [file Table_1.DOCX]

Supplementary Material

**No Interactions of Stacked Bt Maize with the Non-Target Aphid *Rhopalosiphum padi* and the Spider Mite *Tetranychus urticae***

Yinghua Shu, Jörg Romeis, Michael Meissle*

*** Correspondence:** Michael Meissle: [michael.meissle@agroscope.admin.ch](mailto:michael.meissle@agroscope.admin.ch)

# Supplementary Data

The original data used for statistics and the figure are deposited as supplementary material to the main article.

# Supplementary Figures and Tables


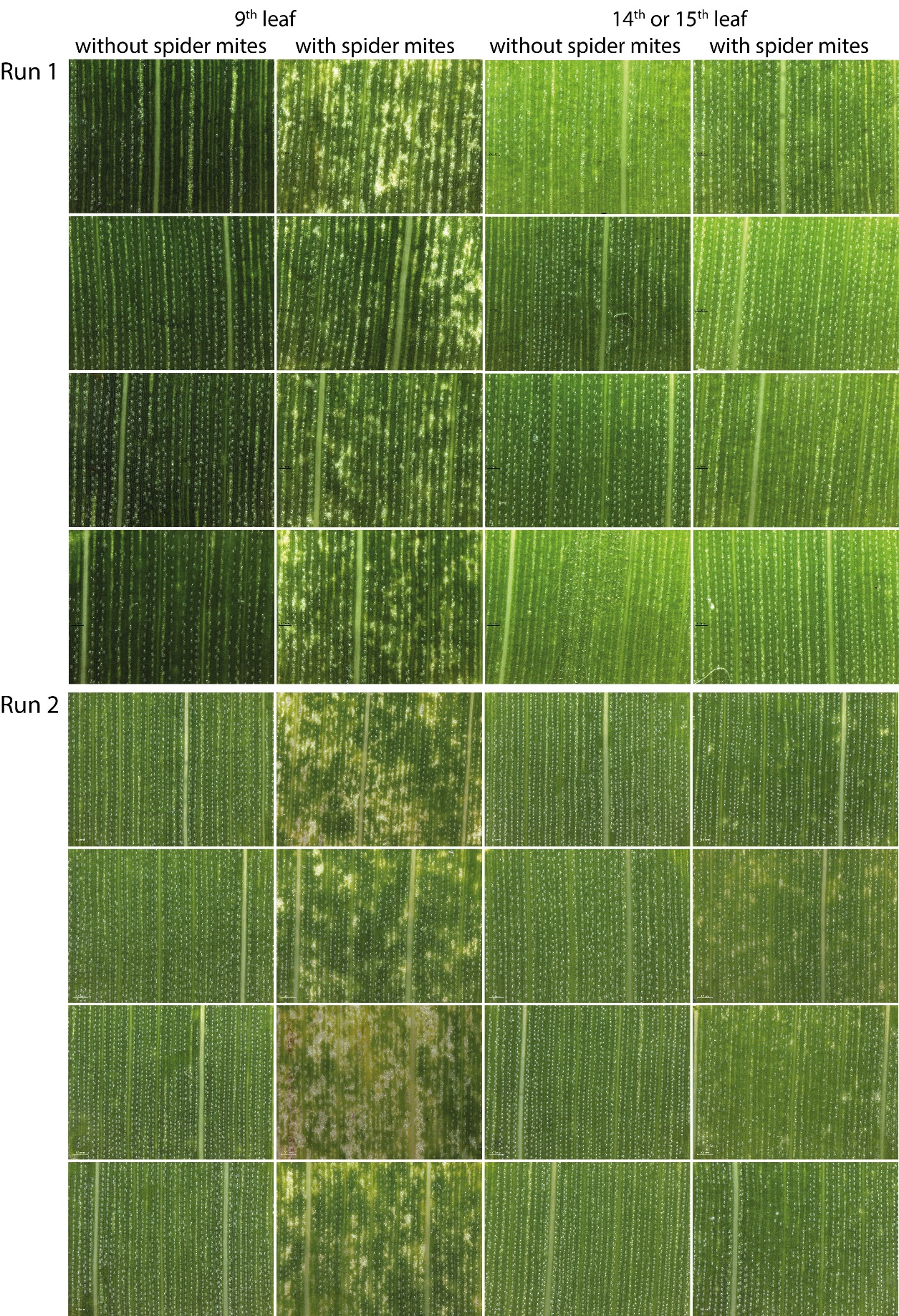


**Figure S1:** Photographs of leaves from plants infested with spider mites three weeks earlier or uninfested plants. Photographs on the upper part of the panel were taken after the first run of the experiment, those in the bottom part after the second run.

**Table S1:** Statistical tests performed with the herbivore life table data. Treatments were SmartStax maize (Bt) or the nearest conventional line (non-Bt), run represents the two repetitions of each experiment in time, and leaf represents the leaf where the clip cage was placed in the aphid experiment or from which discs were cut in the spider mite experiment (leaf 5, 6, or 7 counted from the bottom). Plant was included as a random factor in the GLMM and LMM models, because 3 clip cages were placed on each plant or 3 leaf discs were collected from each plant.

|  | Parameter | Model | Factor | Statistic | p-value |
| --- | --- | --- | --- | --- | --- |
| *Rhopalosiphum padi* | |  |  |  |  |
|  | Nymphal development time | GLMM^1^ | Treatment | 0.70 | 0.40 |
|  |  |  | Run | 10.09 | 0.001 |
|  |  |  | Leaf | 1.01 | 0.60 |
|  |  |  | Treatment:Run | 0.005 | 0.95 |
|  |  |  | Treatment:Leaf | 1.55 | 0.46 |
|  |  |  | Run:Leaf | 0.59 | 0.75 |
|  |  |  | Treatment:Run:Leaf | 0.28 | 0.87 |
|  | Adult longevity | GLMM^1^ | Treatment | 0.006 | 0.94 |
|  |  |  | Run | 19.42 | <0.0001 |
|  |  |  | Leaf | 4.99 | 0.08 |
|  |  |  | Treatment:Run | 0.76 | 0.38 |
|  |  |  | Treatment:Leaf | 11.54 | 0.003 |
|  |  |  | Run:Leaf | 6.02 | 0.049 |
|  |  |  | Treatment:Run:Leaf | 2.22 | 0.33 |
|  | Total fecundity | LMM^2^ | Treatment | 0.49 | 0.48 |
|  |  |  | Run | 18.70 | <0.0001 |
|  |  |  | Leaf | 0.08 | 0.96 |
|  |  |  | Treatment:Run | 0.49 | 0.49 |
|  |  |  | Treatment:Leaf | 0.27 | 0.87 |
|  |  |  | Run:Leaf | 2.77 | 0.25 |
|  |  |  | Treatment:Run:Leaf | 4.70 | 0.10 |
|  | Total longevity | Log-rank^4^ | Treatment | 0 | 0.85 |
| *Tetranychus urticae* | |  |  |  |  |
|  | Egg hatching time | GLM^3^ | Treatment | 0.05 | 0.82 |
|  |  |  | Run | 0.005 | 0.95 |
|  |  |  | Treatment:Run | 0.05 | 0.82 |
|  | Sex | GLM^3^ | Treatment | 0.86 | 0.35 |
|  |  |  | Run | 3.15 | 0.08 |
|  |  |  | Treatment:Run | 1.51 | 0.22 |
|  | Nymphal development time | GLMM^1^ | Treatment | 0.11 | 0.74 |
|  |  |  | Run | 0.03 | 0.87 |
|  |  |  | Leaf | 0.15 | 0.93 |
|  |  |  | Treatment:Run | 0.27 | 0.60 |
|  |  |  | Treatment:Leaf | 0.13 | 0.94 |
|  |  |  | Run:Leaf | 0.03 | 0.99 |
|  |  |  | Treatment:Run:Leaf | 0.34 | 0.84 |
|  | Female longevity | GLMM^1^ | Treatment | 0.02 | 0.90 |
|  |  |  | Run | 58.97 | <0.0001 |
|  |  |  | Leaf | 0.07 | 0.97 |
|  |  |  | Treatment:Run | 1.87 | 0.17 |
|  |  |  | Treatment:Leaf | 0.13 | 0.98 |
|  |  |  | Run:Leaf | 4.51 | 0.11 |
|  |  |  | Treatment:Run:Leaf | 11.26 | 0.004 |
|  | Total fecundity | LMM^2^ | Treatment | 0.08 | 0.78 |
|  |  |  | Run | 36.30 | <0.0001 |
|  |  |  | Leaf | 1.27 | 0.53 |
|  |  |  | Treatment:Run | 0.001 | 0.97 |
|  |  |  | Treatment:Leaf | 0.52 | 0.77 |
|  |  |  | Run:Leaf | 2.32 | 0.31 |
|  |  |  | Treatment:Run:Leaf | 10.56 | 0.005 |
|  | Total longevity | Log-rank^4^ | Treatment | 0.40 | 0.51 |

^1^ generalized linear mixed-effects model (Poisson family) with fixed factors (treatment, run, and leaf) in a full factorial design and random factor (plant), using orthogonal contrasts, followed by ANOVA with type III sum of squares. Reported is the Wald Chi-Square statistic.

^2^ linear mixed effects model with fixed factors (treatment, run, leaf) in a full factorial design and random factor (plant), using orthogonal contrasts, followed by ANOVA with type III sum of squares. Reported is the Wald Chi-Square statistic.

^3^ generalized linear model (Poisson family for hatching time, binomial family with logit link function for sex) with fixed factors (treatment * run) in a full factorial design using orthogonal contrasts, followed by ANOVA with type III sum of squares. Reported is the LR Chi-Square statistic.

^4^ log-rank test based on survival estimates from egg (spider mites) or nymph (aphids) to adult. Death was defined as event (1) and lost or removed individuals were defined as censored (0). All data were pooled.

**Table S2:** Estimated Cry protein concentrations in leaves of SmartStax maize before and after infestation with aphids (*Rhopalosiphum padi*) or spider mites (*Tetranychus urticae*). Given is the number of samples (N), mean concentration (μg/g dry weight), standard error (SE), and the 95% confidence interval (CI). Time indicates if the sample was taken before infestation or three weeks after infestation; position indicates if the 9^th^ leaf was sampled or the first fully expanded top leaf; infested indicates if the plant was infested with herbivores or was designated to be infested (yes) or if it remained uninfested (no); run indicates the experimental repetition in time. Data are plotted in Figure 1 of the main manuscript.

|  | Cry protein | Time | Position | Run | Infested | N | Mean | SE | CI |
| --- | --- | --- | --- | --- | --- | --- | --- | --- | --- |
| *Rhopalosiphum padi* | | |  |  |  |  |  |  |  |
|  | Cry1A.105 | Before | 9th | 1 | No | 10 | 42.33 | 1.395 | 3.156 |
|  |  |  |  |  | Yes | 10 | 44.60 | 3.607 | 8.160 |
|  |  |  |  | 2 | No | 12 | 27.56 | 1.248 | 2.748 |
|  |  |  |  |  | Yes | 12 | 28.63 | 2.088 | 4.596 |
|  |  | After | 9th | 1 | No | 10 | 21.05 | 0.821 | 1.856 |
|  |  |  |  |  | Yes | 10 | 22.40 | 3.413 | 7.721 |
|  |  |  |  | 2 | No | 12 | 26.01 | 2.283 | 5.024 |
|  |  |  |  |  | Yes | 12 | 33.04 | 3.978 | 8.755 |
|  |  |  | Top | 1 | No | 10 | 15.13 | 0.729 | 1.650 |
|  |  |  |  |  | Yes | 10 | 19.23 | 1.885 | 4.264 |
|  |  |  |  | 2 | No | 12 | 13.67 | 0.814 | 1.793 |
|  |  |  |  |  | Yes | 12 | 21.96 | 4.718 | 10.385 |
|  | Cry1F | Before | 9th | 1 | No | 10 | 30.79 | 0.958 | 2.167 |
|  |  |  |  |  | Yes | 10 | 34.57 | 1.910 | 4.322 |
|  |  |  |  | 2 | No | 12 | 13.15 | 0.530 | 1.167 |
|  |  |  |  |  | Yes | 12 | 12.91 | 0.970 | 2.135 |
|  |  | After | 9th | 1 | No | 10 | 25.34 | 1.040 | 2.352 |
|  |  |  |  |  | Yes | 10 | 23.98 | 3.369 | 7.621 |
|  |  |  |  | 2 | No | 12 | 12.40 | 0.925 | 2.037 |
|  |  |  |  |  | Yes | 12 | 20.70 | 1.689 | 3.718 |
|  |  |  | Top | 1 | No | 10 | 14.00 | 1.084 | 2.453 |
|  |  |  |  |  | Yes | 10 | 17.37 | 1.557 | 3.523 |
|  |  |  |  | 2 | No | 12 | 6.20 | 0.319 | 0.702 |
|  |  |  |  |  | Yes | 12 | 8.81 | 1.369 | 3.014 |
|  | Cry2Ab2 | Before | 9th | 1 | No | 10 | 127.44 | 6.162 | 13.940 |
|  |  |  |  |  | Yes | 10 | 131.65 | 6.957 | 15.738 |
|  |  |  |  | 2 | No | 12 | 132.96 | 7.138 | 15.710 |
|  |  |  |  |  | Yes | 12 | 146.47 | 10.074 | 22.172 |
|  |  | After | 9th | 1 | No | 10 | 187.99 | 9.252 | 20.929 |
|  |  |  |  |  | Yes | 10 | 94.83 | 16.294 | 36.860 |
|  |  |  |  | 2 | No | 12 | 153.94 | 14.264 | 31.396 |
|  |  |  |  |  | Yes | 12 | 125.54 | 11.128 | 24.493 |
|  |  |  | Top | 1 | No | 10 | 99.82 | 6.441 | 14.570 |
|  |  |  |  |  | Yes | 10 | 98.65 | 10.498 | 23.748 |
|  |  |  |  | 2 | No | 12 | 76.11 | 4.104 | 9.033 |
|  |  |  |  |  | Yes | 12 | 62.08 | 11.031 | 24.279 |
|  | Cry3Bb1 | Before | 9th | 1 | No | 10 | 38.19 | 1.806 | 4.085 |
|  |  |  |  |  | Yes | 10 | 40.70 | 4.196 | 9.491 |
|  |  |  |  | 2 | No | 12 | 92.58 | 10.762 | 23.688 |
|  |  |  |  |  | Yes | 12 | 51.80 | 5.729 | 12.610 |
|  |  | After | 9th | 1 | No | 10 | 20.77 | 1.089 | 2.464 |
|  |  |  |  |  | Yes | 10 | 30.03 | 5.859 | 13.254 |
|  |  |  |  | 2 | No | 12 | 42.42 | 4.513 | 9.933 |
|  |  |  |  |  | Yes | 12 | 54.89 | 11.735 | 25.828 |
|  |  |  | Top | 1 | No | 10 | 19.86 | 0.791 | 1.789 |
|  |  |  |  |  | Yes | 10 | 28.19 | 3.936 | 8.903 |
|  |  |  |  | 2 | No | 12 | 42.44 | 7.017 | 15.444 |
|  |  |  |  |  | Yes | 12 | 97.39 | 26.947 | 59.310 |
|  | Cry34Ab1 | Before | 9th | 1 | No | 10 | 37.98 | 2.211 | 5.003 |
|  |  |  |  |  | Yes | 10 | 41.53 | 2.773 | 6.273 |
|  |  |  |  | 2 | No | 12 | 31.29 | 1.092 | 2.404 |
|  |  |  |  |  | Yes | 12 | 33.24 | 2.303 | 5.068 |
|  |  | After | 9th | 1 | No | 10 | 107.46 | 5.156 | 11.663 |
|  |  |  |  |  | Yes | 10 | 93.64 | 11.325 | 25.619 |
|  |  |  |  | 2 | No | 12 | 81.53 | 7.255 | 15.968 |
|  |  |  |  |  | Yes | 12 | 112.05 | 9.013 | 19.837 |
|  |  |  | Top | 1 | No | 10 | 45.42 | 1.411 | 3.192 |
|  |  |  |  |  | Yes | 10 | 61.93 | 4.481 | 10.137 |
|  |  |  |  | 2 | No | 12 | 39.91 | 1.597 | 3.515 |
|  |  |  |  |  | Yes | 12 | 62.30 | 9.157 | 20.154 |
| *Tetranychus urticae* | | |  |  |  |  |  |  |  |
|  | Cry1A.105 | Before | 9th | 1 | No | 12 | 45.45 | 4.705 | 10.355 |
|  |  |  |  |  | Yes | 12 | 46.08 | 4.053 | 8.920 |
|  |  |  |  | 2 | No | 13 | 57.45 | 3.035 | 6.612 |
|  |  |  |  |  | Yes | 13 | 56.70 | 2.091 | 4.555 |
|  |  | After | 9th | 1 | No | 12 | 36.50 | 1.779 | 3.915 |
|  |  |  |  |  | Yes | 12 | 50.25 | 4.091 | 9.004 |
|  |  |  |  | 2 | No | 13 | 94.47 | 5.653 | 12.317 |
|  |  |  |  |  | Yes | 13 | 77.56 | 4.652 | 10.135 |
|  |  |  | Top | 1 | No | 12 | 29.50 | 1.805 | 3.972 |
|  |  |  |  |  | Yes | 12 | 31.68 | 3.802 | 8.368 |
|  |  |  |  | 2 | No | 13 | 52.25 | 5.135 | 11.188 |
|  |  |  |  |  | Yes | 13 | 43.12 | 3.822 | 8.328 |
|  | Cry1F | Before | 9th | 1 | No | 12 | 12.51 | 0.712 | 1.568 |
|  |  |  |  |  | Yes | 12 | 10.42 | 0.702 | 1.545 |
|  |  |  |  | 2 | No | 13 | 20.91 | 1.378 | 3.002 |
|  |  |  |  |  | Yes | 13 | 19.52 | 0.733 | 1.596 |
|  |  | After | 9th | 1 | No | 12 | 13.23 | 1.090 | 2.400 |
|  |  |  |  |  | Yes | 12 | 22.80 | 1.805 | 3.973 |
|  |  |  |  | 2 | No | 13 | 31.68 | 1.348 | 2.936 |
|  |  |  |  |  | Yes | 13 | 32.18 | 2.623 | 5.715 |
|  |  |  | Top | 1 | No | 12 | 8.15 | 0.306 | 0.675 |
|  |  |  |  |  | Yes | 12 | 9.00 | 0.410 | 0.902 |
|  |  |  |  | 2 | No | 13 | 19.65 | 1.047 | 2.281 |
|  |  |  |  |  | Yes | 13 | 18.14 | 1.287 | 2.803 |
|  | Cry2Ab2 | Before | 9th | 1 | No | 12 | 145.00 | 27.866 | 61.334 |
|  |  |  |  |  | Yes | 12 | 120.12 | 12.999 | 28.610 |
|  |  |  |  | 2 | No | 13 | 63.05 | 3.125 | 6.808 |
|  |  |  |  |  | Yes | 13 | 66.03 | 4.618 | 10.062 |
|  |  | After | 9th | 1 | No | 12 | 171.27 | 19.314 | 42.510 |
|  |  |  |  |  | Yes | 12 | 144.63 | 27.588 | 60.720 |
|  |  |  |  | 2 | No | 13 | 113.67 | 9.190 | 20.024 |
|  |  |  |  |  | Yes | 13 | 114.99 | 6.479 | 14.116 |
|  |  |  | Top | 1 | No | 12 | 97.19 | 7.655 | 16.849 |
|  |  |  |  |  | Yes | 12 | 104.94 | 14.321 | 31.520 |
|  |  |  |  | 2 | No | 13 | 207.41 | 11.340 | 24.709 |
|  |  |  |  |  | Yes | 13 | 229.10 | 16.072 | 35.017 |
|  | Cry3Bb1 | Before | 9th | 1 | No | 12 | 152.99 | 25.091 | 55.224 |
|  |  |  |  |  | Yes | 12 | 123.83 | 17.285 | 38.044 |
|  |  |  |  | 2 | No | 13 | 135.97 | 8.167 | 17.794 |
|  |  |  |  |  | Yes | 13 | 145.09 | 7.779 | 16.949 |
|  |  | After | 9th | 1 | No | 12 | 93.36 | 10.100 | 22.231 |
|  |  |  |  |  | Yes | 12 | 160.87 | 34.087 | 75.025 |
|  |  |  |  | 2 | No | 13 | 155.27 | 6.573 | 14.320 |
|  |  |  |  |  | Yes | 13 | 144.95 | 5.667 | 12.347 |
|  |  |  | Top | 1 | No | 12 | 105.41 | 9.371 | 20.625 |
|  |  |  |  |  | Yes | 12 | 166.75 | 17.043 | 37.512 |
|  |  |  |  | 2 | No | 13 | 156.41 | 8.567 | 18.667 |
|  |  |  |  |  | Yes | 13 | 142.50 | 7.375 | 16.069 |
|  | Cry34Ab1 | Before | 9th | 1 | No | 12 | 48.98 | 4.130 | 9.089 |
|  |  |  |  |  | Yes | 12 | 43.86 | 3.335 | 7.340 |
|  |  |  |  | 2 | No | 13 | 73.59 | 6.746 | 14.699 |
|  |  |  |  |  | Yes | 13 | 76.01 | 6.054 | 13.191 |
|  |  | After | 9th | 1 | No | 12 | 98.92 | 9.039 | 19.894 |
|  |  |  |  |  | Yes | 12 | 193.97 | 11.039 | 24.296 |
|  |  |  |  | 2 | No | 13 | 189.38 | 7.325 | 15.960 |
|  |  |  |  |  | Yes | 13 | 252.34 | 8.997 | 19.603 |
|  |  |  | Top | 1 | No | 12 | 44.25 | 1.749 | 3.849 |
|  |  |  |  |  | Yes | 12 | 60.25 | 3.226 | 7.100 |
|  |  |  |  | 2 | No | 13 | 134.18 | 6.343 | 13.820 |
|  |  |  |  |  | Yes | 13 | 141.91 | 9.742 | 21.227 |
